# Marek’s Disease Virus Telomeric Integration Profiles of Neoplastic Host Tissues Reveal Unbiased Chromosomal Selection and Loss of Cellular Diversity during Tumorigenesis

**DOI:** 10.3390/genes12101630

**Published:** 2021-10-17

**Authors:** Marla C. Glass, Justin M. Smith, Hans H. Cheng, Mary E. Delany

**Affiliations:** 1 Department of Surgery, Stanford University School of Medicine, Stanford, CA 94305, USA; 2 Department of Animal Science, University of California Davis, Davis, CA 95616, USA; jmcsmith@ucdavis.edu (J.M.S.); medelany@ucdavis.edu (M.E.D.); 3 Avian Disease and Oncology Laboratory, United States Department of Agriculture, Agricultural Research Service, East Lansing, MI 48823, USA; hcheng@msu.edu

**Keywords:** herpesvirus, Marek’s disease virus (MDV), viral integration, chicken, avian genomics, FISH, telomere, lymphoma, viral oncogenesis, cytogenomics

## Abstract

The avian α-herpesvirus known as Marek’s disease virus (MDV) linearly integrates its genomic DNA into host telomeres during infection. The resulting disease, Marek’s disease (MD), is characterized by virally-induced lymphomas with high mortality. The temporal dynamics of MDV-positive (MDV^+^) transformed cells and expansion of MD lymphomas remain targets for further understanding. It also remains to be determined whether specific host chromosomal sites of MDV telomere integration confer an advantage to MDV-transformed cells during tumorigenesis. We applied MDV-specific fluorescence in situ hybridization (MDV FISH) to investigate virus-host cytogenomic interactions within and among a total of 37 gonad lymphomas and neoplastic splenic samples in birds infected with virulent MDV. We also determined single-cell, chromosome-specific MDV integration profiles within and among transformed tissue samples, including multiple samples from the same bird. Most mitotically-dividing cells within neoplastic samples had the cytogenomic phenotype of ‘MDV telomere-integrated only’, and tissue-specific, temporal changes in phenotype frequencies were detected. Transformed cell populations composing gonad lymphomas exhibited significantly lower diversity, in terms of heterogeneity of MDV integration profiles, at the latest stages of tumorigenesis (>50 days post-infection (dpi)). We further report high interindividual and lower intraindividual variation in MDV integration profiles of lymphoma cells. There was no evidence of integration hotspots into a specific host chromosome(s). Collectively, our data suggests that very few transformed MDV^+^ T cell populations present earlier in MDV-induced lymphomas (32–50 dpi), survive, and expand to become the dominant clonal population in more advanced MD lymphomas (51–62 dpi) and establish metastatic lymphomas.

## 1. Introduction

MDV is a ubiquitous oncogenic avian α-herpesvirus that induces MD, which is characterized by visceral tumors and nerve enlargement in susceptible host chicken populations. The severe form of MD often leads to mortality in susceptible birds. MD is controlled by biosecurity, widespread vaccination, and selection for genetic resistance. MDV-infected birds serve as an important disease model system for human oncogenic herpesvirus-associated health conditions. Furthermore, chicken MD research is of critical, global agricultural importance and directly impacts food production, animal health and welfare, and economic sustainability of commercial production systems.

Early in MDV infection, oncogenic strains transition from a cytolytic replicative stage consisting of circularized extra-chromosomal MDV in host cells, peaking around 1 week after infection, to a latent infection stage wherein the virus is no longer replicating and evades host immune detection and responses [1,2,3,4,5,6]. MDV latency has significant temporal overlap with viral linear-integration events into the telomeres of host chromosome [7,8] and a reduction in the quantity of extra-chromosomal, circularized viral genomes [9,10]. 

A major feature of MD, particularly when induced by newly-emerged pathogenic field MDV strains, is the development of fatal lymphomas in the visceral organs starting as early as 2 to 3 weeks post-infection [11,12,13,14,15,16]. MD lymphomas are typically composed of transformed, MDV^+^ CD4^+^ T lymphocytes and other host cells that have infiltrated the lymphoma tissue [12,17,18,19,20]. MDV-encoded *Meq* oncogene expression is essential for virus-induced cellular transformation, while viral telomerase RNA (vTR) expression also contributes to this process [21,22,23,24,25]. Additionally, telomerase activity and telomere biology are associated with malignant transformation [26,27,28]. The majority (>90%) of mitotically-active lymphocytes within MD tumor tissues have an MDV telomere-integrated only phenotype [8,10], in which MDV concatemers have linearly-integrated into one or several host telomeres and the chromosome-associated, circular form of MDV (indicative of lytically replicating virus) is absent [10]. Viral telomeric repeats within the MDV genome is critical to its ability to integrate into the host telomeres [29,30], whereas the presence of the viral TR gene is not [31]. Both pathogenic (oncogenic) MDV and apathogenic MD vaccine strains integrate into chicken telomeres shortly after infection (i.e., within 24 h) [7,31]; however, the telomere-integrated only phenotype is unique to oncogenic MDV infections [31] and characteristic of a latently-infected and/or transformed cell population [8,9,10]. MDV strains that cannot integrate into host telomeres and/or transition to an MDV telomere-integrated only phenotype in host T lymphocytes, do not induce lymphomas [7,23,29,31]. For example, *Meq*-deleted (ΔMeq) MDV infection does not induce the MDV integrated only cellular phenotype, and does not establish a latent infection nor induce host T cell transformation [7,32]. Although it remains unknown how MDV integration specifically contributes to oncogenesis, the cellular events of viral integration and latency are linked temporally and interrelated.

MD lymphoma cellular origins and clonality are complicated to evaluate given the mixed cellular composition within transformed tissues, which commonly contain populations of transformed MDV^+^ T lymphocytes as well as tumor-infiltrating immune cells. Prior studies indicated that late-stage MD lymphomas in host birds can be either monoclonal or polyclonal based on single-cell, host chromosomes-specific MDV integration profiles [8]. Notably, greater heterogeneity of MDV integration profiles were observed within earlier stage (21 days post-infection (dpi)) lymphomas, whereas lymphomas from later after infection (61 and 73 dpi) indicated more homogeneity [7]. In other work, repertoire sequencing and spectratyping of the T cell receptor β chain of transformed T lymphocytes from de novo MDV-induced lymphomas indicated monoclonality [18]. It is unknown whether the specific host chromosomal site(s) of MDV telomeric-integration contribute to positive selection for an MDV-transformed T lymphocyte during tumorigenesis. In this hypothetical setting, MDV-infected T lymphocytes with advantageous viral integration profiles would demonstrate increased probabilities of establishing an advanced, monoclonal MD lymphoma(s) that persists later in the course of the disease.

In humans, a number of viruses are highly associated with tumor development and a subset of these resemble MDV infection and MD progression in chickens through numerous features of their viral genome structure and/or virus-induced disease pathogenesis [33,34,35]. Specifically, Epstein-Barr virus (EBV), hepatitis B virus, human papilloma virus-18 and human herpesvirus-6 (HHV-6) are associated with a number of human cancers [36,37,38,39,40,41,42,43]. Furthermore, HHV-6 integrates into human telomeric DNA and contains human telomeric repeats within its genome [44,45,46], while EBV integrates into the host genomic DNA in a subset of EBV-associated cancers [47,48,49,50,51,52,53]. The similarities between MDV and human oncogenic viruses, particularly herpesviruses, highlight the importance of chicken as a medical model organism in the study of human virus-induced diseases and cancers [35,54].

Further insight into the MDV and host DNA interactions, including patterns of telomeric integration sites, and phenotypes in MDV oncogenically-transformed host T lymphocyte populations within and across host birds may help understand underlying processes in herpesvirus-induced tumorigenesis and pathogenesis, and evaluate intra- and inter-individual variation in these processes. Here, we investigated MDV-host interactions at the level of the genome within and among T cell lymphomas found in gonads and spleens in birds infected with JM/102W, an oncogenic MDV strain [55,56]. MDV FISH was applied to assess of cellular MDV phenotypes in these virally transformed tissue samples. Furthermore, the same method was utilized with host chromosome-specific DNA labeling to generate comprehensive MDV integration profiles, with single-cell resolution, for gonad lymphoma and splenic tissues both within and across genetically defined and uniform experimental host birds. MDV-FISH indicated that most mitotically-dividing cell populations within gonad lymphomas and splenic samples after viral infection have the MDV telomere-integrated only phenotype. Additionally, virus-host phenotypes of these populations demonstrated tissue-specific, temporal changes. Through analyses of host chromosome-specific MDV integration profiles for MD gonad lymphomas, we discovered that the transformed-cell populations composing lymphomas exhibit significantly lower diversity at later stages of tumorigenesis (collected at >50 dpi). We also observed a heterogenous mixture of integration profiles between gonad lymphomas from different host birds and, conversely, similar intra-individual profiles for MDV-transformed tissue samples within host birds. Furthermore, there was no evidence for viral integration ‘hotspots’ amongst host chromosomes within our MDV infection model system. By combining our findings with prior data involving distinct oncogenic MDV strains, host bird genotypes and MDV-transformed host tissues, we generate a more comprehensive analysis of the cellular phenotypes and processes that are connected to the development of MDV-associated cancer.

## 2. Materials and Methods

### 2.1. Genetic Resources and MDV Challenge 

**Figure A1:**
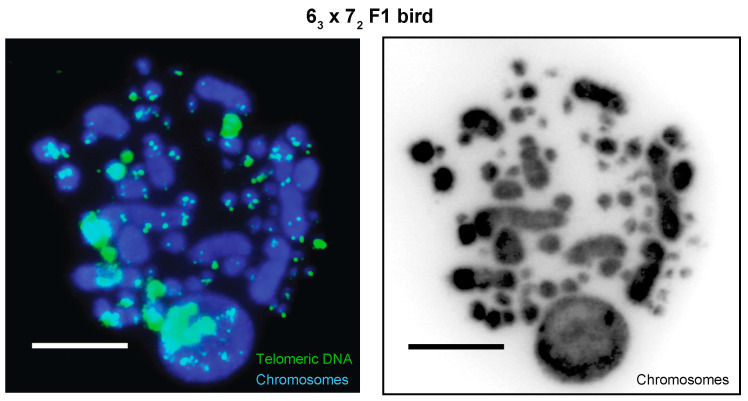
Telomeric DNA profile for mitotically-dividing splenic cells of a line 6_3_ × 7_2_ F_1_ bird indicated by telomere FISH.

Experimental birds were F_1_ progeny from a cross of highly inbred lines (MD-resistant 6_3_ × MD-susceptible 7_2_) from the USDA, ARS, Avian Disease and Oncology Laboratory (ADOL) [57]. Importantly, these experimental F_1_ birds were part of a concomitant study to determine host somatic mutations, including those specific to one line (i.e., genetic resistance status) [58]. The birds and tissue samples that overlapped between our cytogenomic study (data reported herein) and the driver mutation study (data not reported here) is outlined in Supplementary Table S1. The telomeric profiles of experimental F_1_ bird splenic cells matched the canonical chicken telomeric profile (Figure A1). Animals were cared for under approved animal care protocols with all institutional and national guidelines for the appropriate care and use of laboratory animals followed (ADOL animal use protocol no. 13.03). Upon hatching, unvaccinated male and female chicks were subcutaneously injected with 1000 pfu of JM/102W strain MDV [59]. Chicks were hatched and maintained in Horsfall-Bauer isolation chambers at ADOL and checked twice daily for evidence of moribundity with sample collection at such points. 

### 2.2. Tumor Collection and Processing

**Figure 1:**
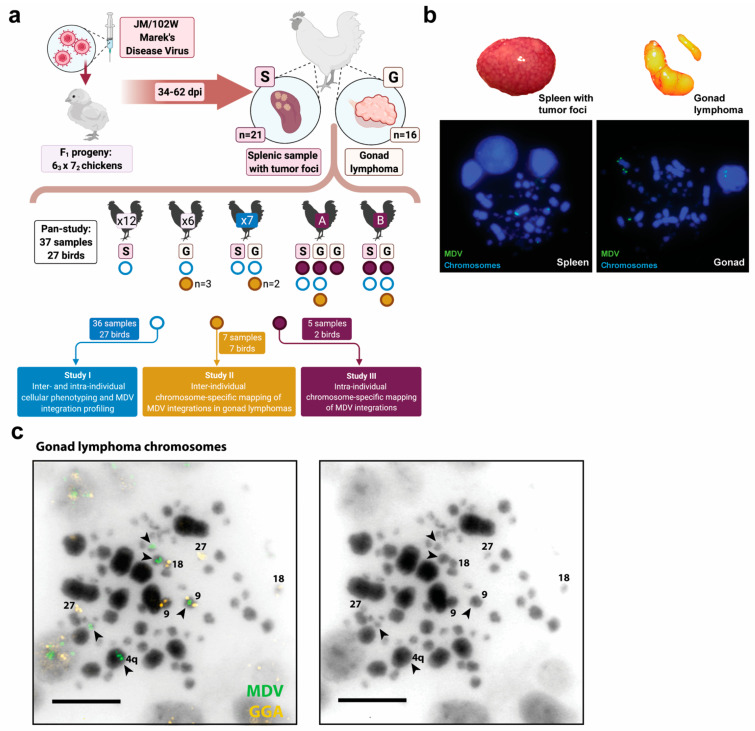
Overview of study design, samples and representative MDV integration status and chromosomes mapping FISH images: (**a**) Experimental workflow and overview of samples and aims for study parts I-III; (**b**) Representative examples of an MDV-infected bird spleen with tumor foci and gonad lymphoma with corresponding transformed host cell-MDV phenotypes of each tissue type. The lower panels show the predominate telomere-integrated only MDV phenotype, which is exclusively comprised of distinct, punctate FISH MDV signals (FITC/green) at the telomeres of host chromosomes (DAPI/blue); (**c**) Representative DAPI-inverted (black/white) FISH image of a transformed host cell from a gonad lymphoma with chromosome-specific mapping of integrated MDV DNA (conducted in study parts II-III). The left panel shows chicken chromosome-specific labelling for GGA 9, 18, and 27 (TRITC/orange) and integrated MDV DNA signals (FITC/green, black arrowheads) at the telomeres of chicken chromosomes 9 (TRITC/orange) and 4q (identified by size and morphology), and three microchromosomes. The right panel shows the same cell without chromosome-specific and MDV labels illustrating the chromosome morphology. Scale bar = 0.5 µM.

Figure 1 provides an overview of the methods, samples and three parts of this cytogenomic study. Briefly, lymphoproliferative foci of the spleen and distinct gonad lymphomas were collected between 34 and 62 dpi from MDV-challenged birds. The spleens with tumor foci samples were estimated to have a range of 1–5% neoplastic cells per sample [60], whereas the gonad lymphomas indicated higher neoplastic cell proportions, with a range of 5–95% neoplastic cells per sample and median of 45%, based on RNAseq analysis [58]. Individual samples were immediately processed into a single-cell suspension by mincing and gentle pipetting and pre-treated for cytogenetics including: colcemid incubation for mitotic arrest, hypotonic treatment to enlarge cells, and cellular fixation to preserve cells as described previously [7]. Fixed cell suspensions were applied to slides by the air-dry method and stored frozen [31,61] until FISH procedures were undertaken. 

### 2.3. Study Design

As Figure 1a illustrates, the study had three parts designed to address distinct aspects of virus-host genomic interaction(s) in MDV^+^ neoplastic tissues within and among individuals and at different timepoints after infection:

Study part I. Thirty-six samples (15 gonad lymphomas and 21 splenic samples) from 27 birds were evaluated in terms of their MDV cellular phenotypes and MDV-integration profiles within and across host nuclei [7,31,62]. MDV integration profiles indicate MDV integration events into host bird individual macrochromosomes (GGA 1-4, Z and W) as well as into intermediate-sized chromosomes and microchromosomes, together as a single category. Both inter-individual (18 birds with one sample from each) and intra-individual (nine birds with two samples from each) sample comparisons were assessed. Some samples were also used for study parts II and III, as denoted in Figure 1a. 

Study part II. Seven gonad lymphomas collected from seven different birds were used to comprehensively evaluate chromosome-specific MDV integration profiles.

Study part III. Five samples (three gonad lymphomas and two splenic samples) from two birds were analyzed to assess intraindividual, chromosome-specific MDV integration profiles. Three samples (two gonad lymphomas and one splenic sample) were from bird ‘A’, and two samples (one gonad lymphoma and one splenic sample) were from bird ‘B.’

### 2.4. Cytogenetic Studies and Analyses

Dual-color FISH with FITC/green labeling for the MDV-specific probe, and DAPI/blue labeling for chromosomes, was employed to broadly evaluate MDV integration profiles and MDV cellular phenotypes in part I of the study. In parts II and III of the study, multicolor FISH was employed, specifically with FITC/green labeling for the MDV-specific probe, TRITC/orange or red labeling for the chromosome-specific probes, and DAPI/blue labeling for chromosomes, to specifically map host bird chromosomes with MDV integrations.

### 2.5. MDV and Chromosome-Specific Probes

The DNA probe utilized for hybridization to the MDV genome was an Md11-strain bacterial artificial chromosome (BAC), Md11gDc1.2 [63]. Chicken chromosome-specific probes used for parts II and II of the study are listed in Supplementary Table S2. Some of the chicken microchromosomes lack published sequence content and, consequently, DNA probes and were not able to be evaluated in MDV integration mapping experiments.

### 2.6. Probe Labeling and Fluorescence In Situ Hybridization (FISH)

Standard labeling and FISH procedures were followed [31,61]. Briefly, the MDV probe was labeled using the Roche DIG-Nick Translation Kit (Millipore Sigma, St. Louis, MO, USA) and hybridized to the mitotic chromosome spreads in the presence of chicken telomeric repeat (TTAGGG_n_) blocking solution known as “cold telo” and an anti-DIG-Fluorescein (Millipore Sigma) employed for a green MDV label (study parts I-III); host bird chromosomes were visualized using Vectashield Mounting Media with DAPI/blue (Abcam, Cambridge, MA, USA).

For study parts II and III, the chromosome-specific probes were labelled using orange-dUTP or red-dUTP (Abbott Molecular, Des Plaines, IL, USA) by the direct Nick Translation Kit (Abbott Molecular). Typically, three chromosome-specific DNA probes were included in the same FISH experiment. The DNA probes co-incorporated into a single experiment were selected based on being able to clearly discriminate their chromosomes targets (i.e., targeted chromosomes with observable size or morphological differences from each other) (Figure 1c).

All images were collected using an Olympus BX41 epifluorescence microscope equipped with an automatic filter wheel (Chroma Technology 82000, DAPI/FITC/TRITC filter set), X-cite 120 Series metal-halide fiber optic lamp and Applied Imaging software (CytoVision version 7.4 GENUS, Leica Biosystems, Buffalo Grove, IL, USA). 

A range of 43 to 108 (mean of 54) gonad-tumor metaphase cell images and a range of 21 to 51 (mean of 45) splenic-tumor metaphase cell images were captured and analyzed. Negative control (no MDV infection) samples were occasionally incorporated into FISH experiments to ensure that the Md11 BAC probe, labeled with FITC, was hybridizing specifically to the MDV genome, as indicated by the absence of FITC signals from all terminal and interstitial telomeres with MDV- (negative control) samples, as described by Robinson and colleagues [8]. 

**Figure 2:**
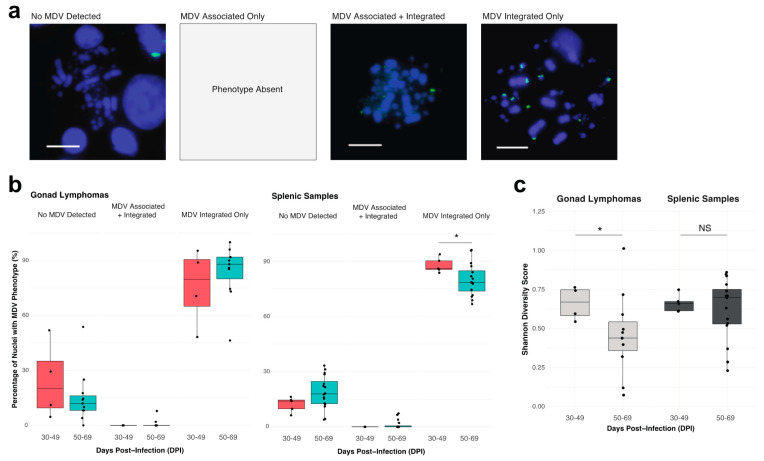
MD gonad lymphomas and splenic samples primarily consist of cells with an MDV telomere-integrated only phenotype and gonadal cell populations exhibit lower diversity in terms of MDV integration profiles at later collection timepoints: (**a**) Representative MDV cellular phenotypes of MD tumors for the oncogenic JM/102W MDV strain, analyzed within study part I. The “No MDV Detected” or null phenotype lacks any evidence for MDV FISH signals associated with or integrated into the host bird chromosomes (DAPI/blue). The “MDV chromosome-associated” phenotype was absent from MDV-transformed tissue samples. The “MDV chromosome-associated/telomere-integrated” phenotype consists of both diffuse chromosome-associated MDV signals surrounding the chromosomes and bright, punctate integrated-MDV signals at the host telomeres. The telomere-integrated only MDV phenotype is exclusively comprised of distinct, punctate FISH MDV signals at the telomeres. Scale bar = 0.5 µM; (**b**) Percentage of cells with detected MDV cellular phenotypes for tissue collection time ranges, 30–49 dpi (orange) versus 50–69 dpi (teal blue), of MD gonad lymphomas (left plot; *n* = 15) and splenic samples (right plot; *n* = 21) in study part I. Line inside box depicts median value, box depicts interquartile range (IQR), and whiskers depict IQR+/− 1.5*IQR. Dots indicate tumor values. * *p* < 0.05; (**c**) Shannon diversity score of MD gonad lymphomas (left plot, light gray; *n* = 15) and splenic samples (right plot, dark gray; *n* = 21) from tissue collection time ranges, 30–49 dpi versus 50–69 dpi in study part I. A higher score indicates a greater diversity, in terms of MDV integration profiles, of the FISH-analyzed cell population in a tissue sample. Line inside the box depicts median value, box depicts interquartile range (IQR), and whiskers depict IQR+/− 1.5*IQR. * *p* < 0.05.

For part I of the study, captured mitotic metaphase cells were categorized as one of four MDV cellular phenotypes as previously described [7,31]; null (no signal), MDV chromosome-associated, MDV chromosome-associated and telomere-integrated, or MDV telomere-integrated only (outlined in Figure 2a). The cells exhibiting the MDV telomere-integrated cellular phenotypes were further analyzed for MDV telomere-integration events into individual macrochromosomes (GGA 1, 2, 3, 4), individual sex chromosomes (Z and W), and the intermediate and micro-chromosomes (GGA 5-38, represented as a single category). The chromosomes were identified based on chromosome sizes and morphological traits [64,65,66,67].

### 2.7. Data Visualization and Statistical Analysis 

Study part I: The percentage of cells in each of the four MDV cellular phenotype categories (outlined in Figure 2a) was derived from FISH analyses of mitotic metaphase cell populations of each MD gonad lymphoma and splenic sample. The percentages of cells within the MDV cellular phenotype categories were compared between tissue collection time ranges (30–49 dpi versus 50–69 dpi) separately for each tissue type (gonad lymphomas and splenic samples) by the Wilcoxon rank sum test with Bonferroni correction. Any differences with a *p*-value of less than or equal to 0.05 were considered statistically significant (denoted by a * symbol).

The Shannon diversity score for each gonad lymphoma and splenic sample was computed by substitution of ‘species’ populations and frequencies with ‘MDV integration’ chromosome sites and frequencies [68]. For each tissue type, the diversity score of samples were compared between the tissue collection time ranges (30–49 dpi versus 50–69 dpi) by the Wilcoxon rank sum test. Any differences with a *p*-value of less than or equal to 0.05 were considered statistically significant (denoted by a * symbol).

Study parts II and III: The observed frequency of MDV integration events for all individual chromosomes (GGA 1-28, 32, Z) across eight gonad lymphomas, two of which were from the same bird, were calculated as chromosomes-specific MDV integration counts divided by total MDV integrations counts across all MDV^+^ nuclei. The observed frequencies were compared by two-factor ANOVA and Tukey’s multiple comparisons of means post-hoc test, with a 95% family-wise confidence interval, in R (https://www.r-project.org/, accessed on 19 September 2021). Expected frequencies of random/unbiased MDV integration was calculated as 1/39 for the autosomal chromosomes (GGA 1-28, 32), 1/78 for the sex chromosome GGA Z in female birds (ZW), and 1/39 for GGA Z in male birds (ZZ). No MDV integration events were detected in the sex chromosome GGA W within sampled tissues in study parts II-III. The expected frequency represented the probability of MDV integration within the distal telomere of a specific or individual chromosome among total chromosomes present in a host nucleus under random or unbiased processes.

The Shannon diversity score for each gonad lymphoma was computed as described above; specifically, through the application of MDV integration frequencies for individual chromosomes (GGA 1-28, 32, Z, W). The diversity score of samples were compared between gonad lymphoma collection time ranges (50–52 dpi versus 60–61 dpi) by the Wilcoxon rank sum test with Bonferroni correction. For all statistical test results, a *p*-value of less than or equal to 0.05 was considered statistically significant.

Cross-study comparison: For 21 MD gonad lymphomas, median MDV integrations per MDV^+^ nuclei for each chromosome (individually for GGA 1, 2, 3, 4, Z) and chromosome category (collectively for macrochromosomes or GGA 1-4, Z; intermediates & microchromosomes or GGA 5-38, W; median adjusted by number of chromosomes in category) were compared by two-factor ANOVA and Tukey’s multiple comparisons of means post-hoc test, with a 95% family-wise confidence interval, in R (https://www.r-project.org/, accessed on 19 September 2021). *p*-values of less than or equal to 0.05 were considered statistically significant. However, no differences in the median MDV integration events at any chromosome or chromosome category were significant.

## 3. Results

### 3.1. Study Part I. Inter- and Intra-Individual MDV Cellular Phenotyping and Integration Profiling in MD Lymphomas from Gonadal and Splenic Tissues

#### 3.1.1. MDV Cellular Phenotyping

In part I of this study, we sought to understand the temporal dynamics of an oncogenic MDV strain (JM/102W) in host cells by studying MDV-transformed samples at timepoints after initial infection (i.e., stage of MD progression). Here, we utilized FISH-based methods to assess phenotype of MDV in host nuclei of virus-transformed chicken gonad lymphomas and spleens with lymphoproliferative foci tissue samples collected between 30 to 69 dpi (Figure 1a,b; see Methods). FISH-evaluated samples were from the same bird (*n* = 18 samples among 9 birds) and from different birds (*n* = 18 samples from 18 birds), to capture both inter- and intra-individual variation. Importantly, our MDV FISH results capture only mitotically-dividing cells within neoplastic tissues. Therefore, these findings should not be assumed to apply to non-dividing and/or peripheral cells of host birds.

We observed three of four previously described MDV phenotypes [7,8,31,62] in the samples: specifically, the MDV null phenotype (no virus), the MDV chromosome-associated/telomere-integrated phenotype and the telomere-integrated-only MDV phenotype (Figure 2a). The MDV chromosome-associated-only phenotype was not observed in either sample type. The predominant phenotype was MDV telomere-integrated-only, which represented 45–99% of analyzed nuclei in tissue samples (Figure 2b). In contrast, the MDV chromosome-associated/-telomere-integrated phenotype represented 0–10% of total nuclei across samples. Interestingly, this phenotype was limited to the samples collected in the later timepoints after infection (50–69 dpi). The temporal dynamics of the MDV null (no virus) and MDV telomere-integrated-only phenotypes notably differed between the tissue sample types (Figure 2b). For MD gonad lymphomas, the proportion of cells without MDV infection (null) was lower in samples collected between 50–69 dpi as compared to those from 30–49 dpi. Conversely, the proportion of MDV telomere-integrated only phenotype cells increased in later timepoint (50–69 dpi) samples. No MDV phenotype changes in gonad lymphomas between sample collection time ranges were statistically significant (*p* > 0.05). For MD splenic samples, the MDV null and MD telomere-integrated only phenotypes showed the opposite trends, in terms of representation within samples collected between 50–69 dpi versus those from 30–49 dpi. Furthermore, the decrease in proportion of cells with the MDV telomere-integrated only phenotype observed in splenic samples from 50–69 dpi (as compared to 30–49 dpi) was statistically significant (*p* < 0.05). 

#### 3.1.2. MDV Integration Profiling

MDV FISH images for 50 or more nuclei in each neoplastic tissue sample were analyzed expressly for viral integration profiles, which assigned frequencies of MDV telomeric integration events to host chromosomes sites (GGA 1, 2, 3, 4, Z, W) and/or groupings (macrochromosomes, intermediate/microchromosomes). We applied Shannon Diversity score, with MDV integration chromosome mapping frequencies (from integration profiling) superimposed as ‘species’ population frequencies within a tissue sample, to evaluate cellular diversity of MD gonad lymphomas and splenic samples. A higher score indicates a more heterogenous or differential mixture of cells, defined by their MDV integration profiles, within a sample. MD gonad lymphomas showed a statistically significant decrease (*p* < 0.05) in diversity score in samples collected between 50–69 dpi as compared those from 30–49 dpi (Figure 2c). The MD splenic samples showed the opposite trend, with an increase in diversity score for samples collected between 50–69 dpi (vs. 30–49 dpi) (Figure 2c). However, this change was not significant (*p* > 0.05).

### 3.2. Study Part II. Inter-Individual Chromosome-Specific Mapping of MDV Integration in Gonad Lymphomas

**Figure 3:**
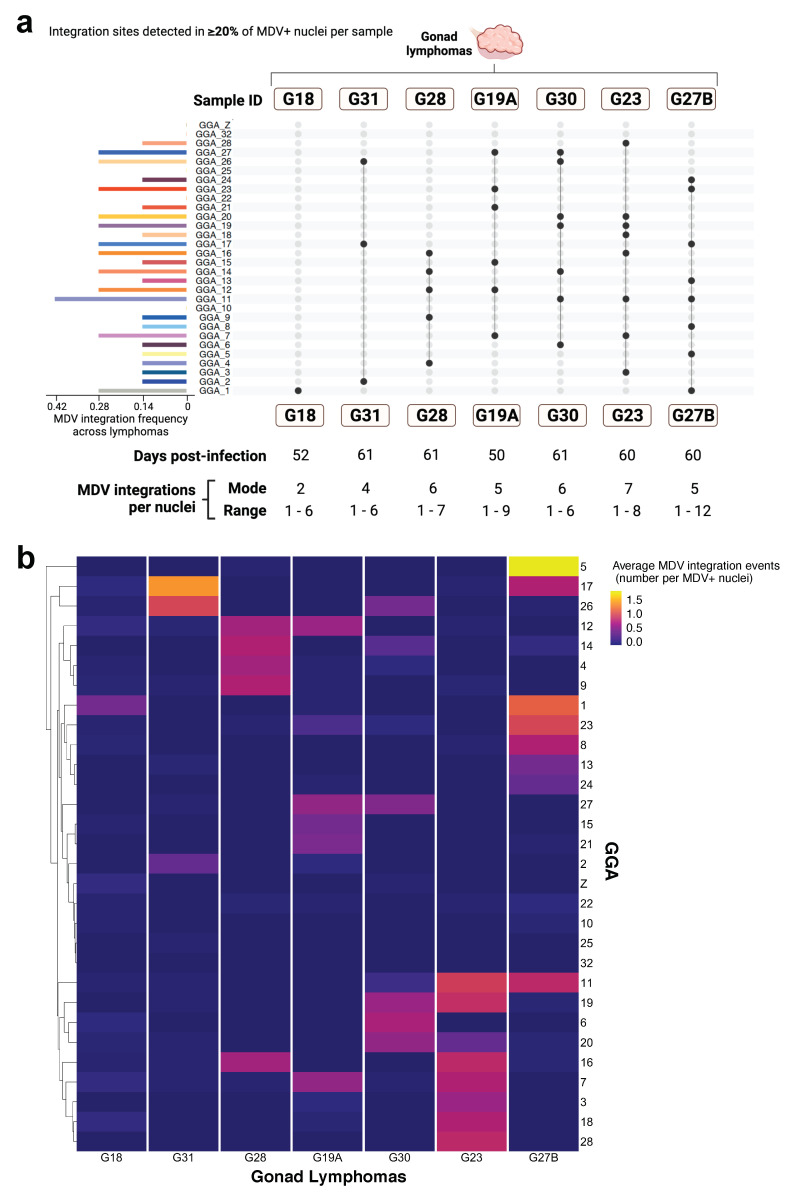
MDV FISH analysis indicates heterogenous inter-individual MDV integration profiles among MD gonad lymphomas from different birds: (**a**) Left bar graph panel shows the frequency of each host chromosome site among MDV integration profiles of gonad lymphomas in study part II. Dot plot (right panel) indicates the profiles of MDV integration at host chromosome sites detected in >20% of MDV-positive (MDV^+^) nuclei from each individual gonad lymphoma (e.g., G18, G31, etc.) of a different MDV-infected bird (study part II; see Figure 1a). Tissue collection timepoints post MDV infection (dpi), and the mode and range of MDV integrations per nuclei are indicated below the dot plot for each lymphoma; (**b**) Average MDV integrations events (as counts) per MDV^+^ nuclei for each host chromosome (*y* axis) for MD gonad lymphomas (*x* axis) from individual birds in study part II. *Y* axis (with host chromosome sites) is organized by unsupervised hierarchical clustering, which means a deep learning algorithm identified a hierarchy of natural groups within the dataset.

Here, we utilized FISH-based methods to assess telomeric integration profiles of the oncogenic JM/102 W strain of MDV into specifically labeled host chicken chromosomes (GGA 1-28, 32, Z, W) of 7 virus-transformed gonad lymphomas collected between 50 to 61 dpi from different birds (Figure 1a,c). Specifically, the single-cell and total counts and frequency of MDV integration at each analyzed chromosome was determined for >50 MDV-positive (MDV^+^) nuclei within each MD gonad lymphoma. The focus of the study design and analysis was inter-individual variation (and shared features) of gonad lymphoma MDV integration profiles. The integration profiles outlined in Figure 3a were defined as host chromosomes with integration events in >20% of MDV-positive (MDV^+^) nuclei in an individual gonad lymphoma (identified by sample labels G18, G31, etc.). The profiles between the gonad lymphoma, collected from different host birds, demonstrated minimal overlap (Figure 3a). Furthermore, we did not identify a preferential MDV integration site among analyzed chromosomes in gonad lymphomas collected between 50–61 dpi with oncogenic JM/102W strain. GGA 11 was the most frequently detected site with MDV telomeric integration events, with a presence in 42% of MDV integration profiles of lymphomas. The average viral integration events per MDV^+^ nuclei (as counts) for each host chromosome were also evaluated, as an alternate method to detect hotspots (Figure 3b). This evaluation indicated sporadic integration sites of MDV amongst distinct MD gonad lymphomas (Figure 3b, samples across *x* axis), as determined by the absence of host chromosomes (*y* axis) with an average integration event value ≥0.5 in >50% of gonad lymphomas. The unsupervised clustering of host chromosome sites further emphasized that hotspots of MDV integration were not present among inter-individual samples. 

### 3.3. Study Part III. Intra-Individual Chromosome-Specific Mapping of MDV Integration in Gonad Lymphomas and Splenic Tissue Samples 

**Figure 4:**
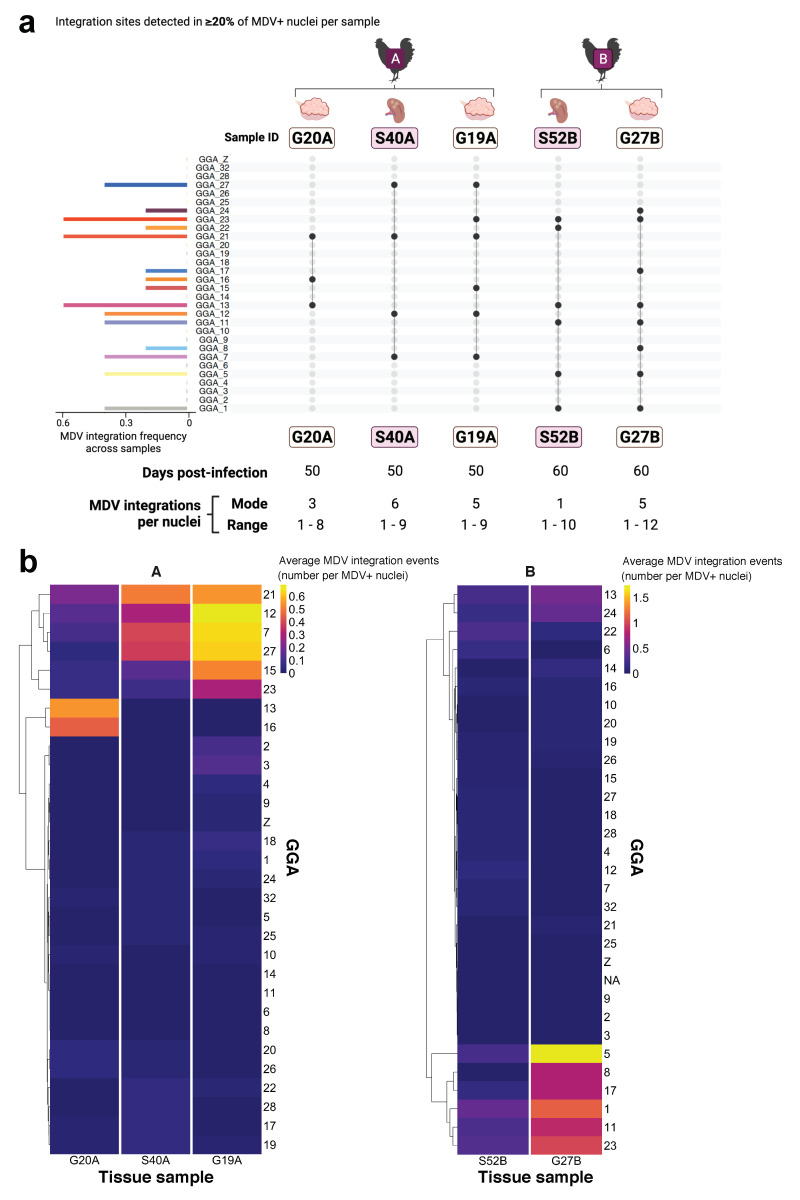
MDV FISH analysis reveals similar intra-individual MDV integration profiles for MD-transformed tissue samples: (**a**) Left bar graph panel depicts frequency of MDV integration for each host chromosome site among MDV integration profiles of transformed tissue samples from two different birds (denoted bird ‘A’ or ‘B’; gonad lymphoma or splenic sample identifier indicated below bird label) in study part III. The dot plot (right panel) depicts the profiles of MDV integration host chromosome sites detected in >20% of MDV^+^ nuclei from gonad lymphomas or splenic samples from the same MD-infected bird (study part III; see Figure 1a). Tissue collection timepoint after MDV infection (dpi), and mode and range of MDV integrations per nuclei reported for each sample below the dot plot panel; (**b**) Average MDV integrations events (as counts) per MDV^+^ nuclei for each host chromosome in MD gonad lymphomas and splenic samples of birds A and B in study part III. *Y* axis (with host chromosome sites) is organized by unsupervised hierarchical clustering.

In this part of the study, we utilized MDV FISH to evaluate the telomeric integration profiles of oncogenic MDV into specific host chicken chromosomes (GGA 1-28, 32, Z, W) in a total of five virus-transformed gonad and splenic samples from two host birds. The relevant samples were collected at 50 dpi from one bird (one splenic sample and two gonad lymphomas from bird ‘A’) and 60 dpi from one bird (one splenic sample and one gonad lymphoma from bird ‘B’; Figure 1a,c). The single-cell and total counts and frequency of MDV integration at each analyzed chromosome was determined for >50 MDV-positive (MDV^+^) nuclei within each MD tissue sample. The focus of the study design and analysis was intra-individual comparisons of splenic sample and gonad lymphoma MDV integration profiles. The profiles shown in Figure 4a were comprised of host chromosomes with virus integration events in >20% of MDV-positive (MDV^+^) nuclei in an individual sample (distinguished by a labels G20A, S40A, etc.). For both analyzed birds A and B, the profiles between the tissues collected from the same host, demonstrated high similarity (Figure 4a). Prevalent MDV integration sites were detected among analyzed chromosomes in ‘sister’ (within same host) tissue samples. Specifically, GGA 21 MDV telomeric integration (3/3 of A samples and 0/2 of B samples) was the predominant in bird ‘A’ samples, followed by GGA 7 (2/3 of A samples and 0/2 of B samples) and 12 (2/3 of A samples and 0/2 of B samples). GGA 1, 5, 11, 13 and 23 integrations (all in 2/2 of B samples; GGA 13 and 23 were also detected in 1/3 of A samples) were enriched in bird ‘B’ samples. 

The average viral integration events per MDV^+^ nuclei (as counts) for each host chromosome were also evaluated, as in study II, revealing several chromosomes enriched for MDV integration amongst distinct tissue samples within the same host bird (Figure 4b, samples across *x* axis). More specifically, the same host chromosomes harbored ≥0.5 MDV integration events on average in multiple or all tissue samples from the same bird. Unsupervised hierarchical clustering of host chromosome sites (*y* axis) further emphasized relatedness of distinct lymphoma samples and the hotspots of MDV integration among intra-individual samples (Figure 4b). This result corroborates with the MDV integration profile findings outlined in Figure 4a. Specific host chromosomes within lymphomas had higher average MDV integration event values, including GGA 5 in bird B samples and GGA 7, 12 and 27 in bird A samples.

### 3.4. Study Parts I–III. Cross-Experiment Analysis of MDV Integration Mapping 

**Figure 5:**
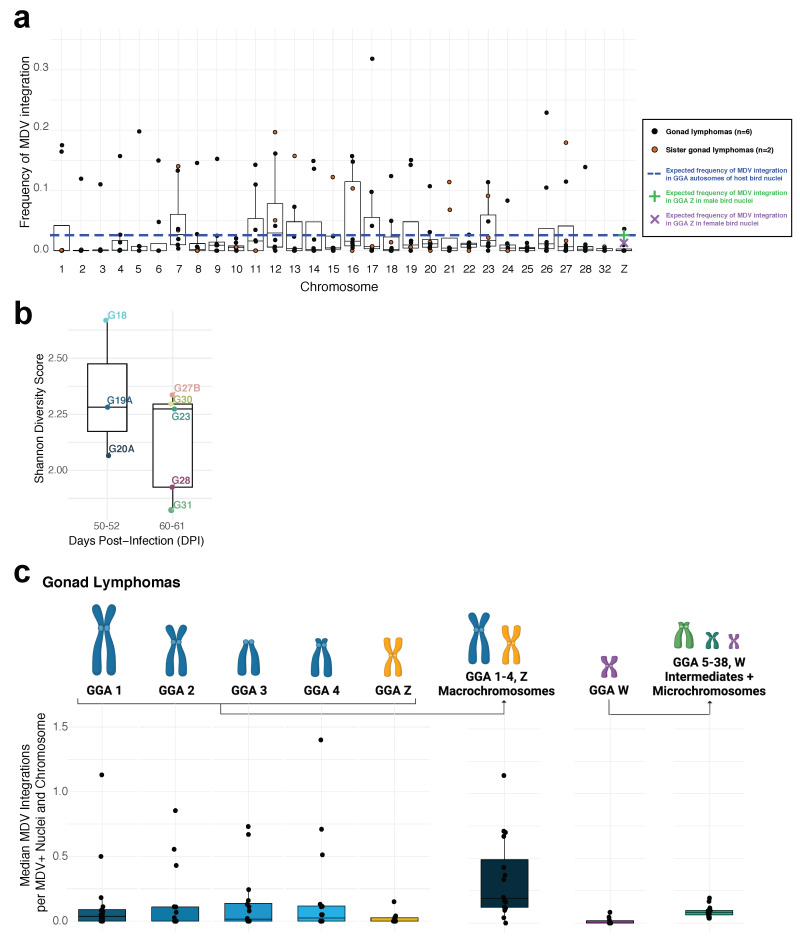
Cross-experiment analysis reveals no statistically significant MDV integration ‘hotspots’ amongst host chromosomes and lower diversity of MDV integration profiles over time in MD gonad lymphomas: (**a**) Observed versus expected frequencies of MDV integration events (*y* axis) mapped to individual host chromosomes (*x* axis) within MD gonad lymphomas (dots) are shown. Boxed line depicts median value, box depicts interquartile range (IQR), and whiskers depict IQR+/− 1.5*IQR. Yellow dots indicate the two gonad lymphomas from the same host MDV-infected bird (bird A; see Figure 1a). The horizontal dashed blue line represents the expected MDV integration frequencies, assuming a random/unbiased process of integration, for autosomal chromosomes (GGA 1-28, 32). The green ‘+’ and purple ‘X’ symbols indicate expected frequency of random MDV integration for GGA Z in male bird nuclei (ZZ) and female bird nuclei (ZW), respectively. Gonad lymphoma data shown are from study parts II-III (see Figure 1a; *n* = 8); (**b**) Shannon diversity score of MD gonad lymphomas collected within the time ranges 50–52 dpi (*n* = 3) versus 60–61 dpi (*n* = 5). Gonad lymphomas are from study parts II and III (see Figure 1a). A higher score indicates a greater diversity, in terms of MDV integration profiles, of the FISH-analyzed cell population within a lymphoma. Boxed line depicts median values, box depicts interquartile range (IQR), and whiskers depict IQR+/− 1.5*IQR. *p* = 0.28; (**c**) Median MDV integrations per MDV^+^ nuclei into each chromosome (individually for GGA 1, 2, 3, 4, Z, W) and chromosome category (collectively for macrochromosomes or GGA 1-4, Z and intermediates + microchromosomes or GGA 5–38, W) in gonad lymphomas from study parts I-III (see Figure 1a; *n* = 21). Medians were adjusted to number of chromosomes in category, where applicable.

Cytogenomic results mapping MDV integrations to GGA 1-28, 32, Z and W from study parts II and III, were used to obtain observed frequencies of MDV integration events mapped to individual host chromosomes for each of eight total MD gonad lymphomas. These observed MDV integration frequencies at each chromosome were compared to the expected frequency of integration assuming these events are completely random or unbiased (i.e., no processes bias where MDV DNA is inserted in host chromosome telomeres) (Figure 5a; see Methods). A host chromosome with evidence of preferential MDV integration among gonad lymphomas was not apparent, as median integration frequencies neither greatly exceeded the ‘expected’ or random integration frequency, nor significantly differed from any other observed integration frequency across analyzed chromosomes (*p* > 0.05). However, GGA 7 and 12 indicated the highest median MDV integration frequencies among lymphomas (Figure 5a). 

As in part I analysis, we utilized MDV integration profiles, which assigned frequencies of viral telomeric integration events to host chromosomes sites (GGA 1-28, 32, Z) to generate an overview of cellular composition in MD gonad lymphomas and assess the cellular heterogeneity or diversity of those samples. We applied Shannon Diversity score, with MDV integration chromosome mapping frequencies (from integration profiling) superimposed as ‘species’ population frequencies, to evaluate cellular diversity of 8 lymphomas. A higher score indicated a more heterogenous or differential mixture of cells, defined by their MDV integration profiles, within a lymphoma. MD gonad lymphomas showed a decreasing trend in diversity score from samples collected at 60–61 dpi versus 50–52 dpi (Figure 5b), but this change was not significant (*p* = 0.28), possibly due to the small gap in sample collection time between the compared groups. 

We also conducted a comprehensive analysis, by pooling data from study parts I-III to obtain the median MDV integrations per MDV^+^ nuclei into individual chromosomes, GGA 1, 2, 3, 4, Z and W, as well as into the chromosome groupings, macrochromosomes (GGA 1-4, Z) and intermediates & microchromosomes (GGA 5-38, W) within 21 total MD gonad lymphomas. The median MDV integrations in the chromosome groupings (macrochromosomes, intermediates & microchromosomes) were adjusted to number of chromosomes in each category, for all gonad lymphomas, to allow for direct comparisons with individual chromosome integration data (GGA 1-4, Z, W). The macrochromosomes, as a group, had a higher median MDV integration per chromosome value as compared to the intermediate-sized chromosomes and microchromosomes group (Figure 5c). However, statistical comparison indicated that there were not significantly elevated MDV integration events amongst individual chromosomes or amongst chromosome groups (*p* > 0.05). 

## 4. Discussion

### 4.1. MD Gonad Lymphomas and Splenic Lymphoproliferative Foci Samples Primarily Consist of Cells with an MDV Telomere-Integrated-Only Phenotype and Demonstrate Unique Temporal Dynamics in MDV Cytogenomic Phenotypes

In this study we demonstrate the oncogenic MDV cytogenomic phenotype dynamics between 30 and 69 dpi for MDV-transformed gonad lymphomas and splenic tissue with lymphoproliferative foci samples. Both sample types consisted of similar proportions of cells with no virus (null), the MDV chromosome-associated/telomere-integrated phenotype and the telomere-integrated only MDV phenotype. The MDV chromosome-associated/telomere-integrated cytogenomic phenotype is indicative of host T cells containing both MDV DNA episomes (chromosome-associated FISH signal) and linearly-integrated MDV DNA (telomere-integrated FISH signals), which is likely transitioning toward or has established a latent MDV infection. The telomere-integrated only phenotype indicates a host cell that is primed for or has undergone MDV-induced oncogenic transformation. In this and prior studies, most mitotically-dividing cells within neoplastic tissues had the MDV telomere-integrated only phenotype [7,62], further supporting the link between this integrated-MDV phenotype and infected T cell transformation. The complete absence of the MDV chromosome-associated only phenotype, characterized by MDV DNA episomes tightly associated with host chromosomes, within the neoplastic splenic and gonad tissues was notable and suggests that there were little to no dividing cells with lytically replicating MDV within these neoplastic tissue samples. However, this finding should not be assumed to also apply to non-dividing and/or circulating cell populations of MDV-infected birds. The lack of this phenotype in transformed tissues collected at least 4 weeks after infection is reasonable given that it is most frequently detected in host tissues early after initial MDV infection (≤14 dpi) and is linked to active viral replication and lytic MDV infection [7,31]. Robinson et al. previously applied FISH methods to evaluate cytogenomic profiles of MD tumors induced by a different MDV strain (GA) in an MD-susceptible host genetic background (inbred host bird line) and reported negligible proportions of cells with the MDV associated-only-phenotype within and across MD lymphoma samples [7]. The minor difference between our phenotyping results may be explained by employment of JM/102W MDV strain infection and/or line 6_3_ × 7_2_ F_1_ host birds in our study, or merely a characteristic of a more prolonged MDV infection. 

### 4.2. MD Gonad Lymphomas Exhibit Significantly Lower Diversity in Terms of MDV Integration Profiles at Later Stages of Tumorigenesis 

A long-standing question in MD progression, is whether MD neoplasm in different tissues persist as diverse mixtures of cancerous cells originating from independently MDV-transformed cells or if, more frequently, these neoplasms become a homogenous population predominated by a single MDV-transformed cell population due to selective forces within the tumor tissue. We sought to address this question by generating MDV integration profiles for the host cells within MD gonad lymphomas and splenic samples with tumor foci and using the metrics from these integration profiles to determine a cellular diversity score for each sample. MD gonad lymphomas showed a significant decrease in diversity score in samples collected between 50–69 dpi versus those from 30–49 dpi. Thus, these lymphoma samples appear to become more homogenous or monoclonal in cellular composition when lymphoma samples are collected after a more prolonged MDV infection. The MD splenic samples showed the opposite trend. The difference in cellular diversity score changes between the sample types is not surprising given the splenic tissues collected at all represented timepoints in the study were comprised of both transformed cluster of cells (tumor foci) and proximal unaffected or normal tissue in the spleen and circulating blood, at varying relative proportions by sampling. Furthermore, RNAseq data for spleens with tumor foci after JM/102W infection suggested a low neoplastic cell frequency (range: 0.5–10%) within these samples [60]. Thus, the observed increase in cellular diversity of later stage MD splenic tissue samples may be explained by technical/experimental variation at tissue sampling and/or biological events unique to this tissue type. Conversely, MD gonad lymphomas were typically large neoplasms that could be easily collected without inclusion of normal/unaffected gonad tissues at most timepoints. Additionally, RNAseq analysis indicated that gonad lymphomas were comprised of more neoplastic cells, with an average estimated purity of 45% (range: 33.2–56.9%) [58]. Thus, the increasing cellular homogeneity seen in these lymphomas is likely driven by selective pressure on the MDV-transformed cell populations as MD progressed.

### 4.3. MDV FISH Analysis Indicates Heterogenous Inter-Individual MDV Integration Profiles among MD Gonad Lymphomas from Different Host Birds

It remains unverified if there are hotspots of MDV telomeric integration events in particular host chromosomes, driven be either the integration mechanism(s) itself or by distinct selective pressures on the MDV-infected and transformed host cells. Prior work demonstrated that MDV integration occurs early, frequently and indiscriminately, in terms of host chromosome sites, after experimental MDV infection of genetically-susceptible host birds [7]. However, latent herpesvirus integration dynamics have not been mapped in MD lymphomas after a more prolonged MDV infection (collected after 21 dpi), besides for the oncogenic GA strain in MD susceptible birds [7]. We evaluated telomeric integration profiles of oncogenic MDV into specifically labeled host chicken chromosomes (GGA 1-28, 32, Z, W) of 7 MD gonad lymphomas from different birds to assess the inter-individual variation in lymphoma MDV integration profiles and determine if integration hotspots in the chicken genome were present. The results indicated that there was no statistically significant preferential MDV integration site among chicken host chromosomes in gonad lymphomas stemming from JM/102W strain MDV infection. Notably, specific host chromosomes within some lymphomas demonstrated elevated MDV integration events (GGA 5 in sample G27B, GGA 17 and 26 in sample G31, etc.), suggestive of a monoclonal origin for most cells in these tissues. Nonetheless, the MDV integration sites enriched in specific lymphomas were largely not detected in other lymphomas. The absence of an MDV integration ‘hotspot’ among MD lymphomas, in this and prior work, suggests that MDV telomeric-integration into a particular host chromosome(s) does not confer a selective advantage to transformed host cells.

### 4.4. MDV FISH Analysis Reveals Similar Intra-Individual MDV Integration Profiles for MDV-Transformed Tissue Samples

Another standing question around MDV-driven transformation of host bird tissues is whether viral integration site preferences emerge amongst MD lymphomas within the same bird. ‘Sister’ lymphomas within the same host have a higher probability of overlap between MDV integration profiles, due to the possibility of transformed cell metastasis and/or analogous selective processes within a shared biological environment. We utilized the MDV telomeric integration profiles of MD gonad lymphomas and splenic samples with tumor foci that developed in the same bird (Figure 1a,c) to assess the intra-individual variation in viral integrations and establish whether neoplastic tissues within an individual bird may have developed from transformed cell metastases. The profiles between the tissues collected from the same bird demonstrated high similarity and prevalent MDV integration sites were detected among host chromosomes in ‘sister’ tissue samples. The notable overlap in profiles between samples collected from distinct tissue sites within the same bird, suggests shared origins of these virus-transformed tissues through neoplastic cell metastases and/or indicates that host cells with particular MDV integration chromosome sites may harbor distinct survival probabilities in a given host environment (i.e., under analogous selective processes), which may cause specific transformed populations to predominate in advanced MD lymphomas. Furthermore, several host chromosomes had markedly higher MDV integration events on average within specific lymphoma samples, possibly indicating more homogenous cell populations at these tissue sites.

### 4.5. Cross-Experiment Analysis Reveals No Significant MDV Integration ‘Hotspots’ Amongst Host Chromosomes and Lower Diversity of MDV Integration Profiles over Time in MD Gonad Lymphomas 

In study parts I, II and III, cytogenomic data was independently used to assess variance in MDV integration profiles between and within host birds. In a final comprehensive analysis, we merged the chromosome-specific MDV integration mapping data from study parts I-III to improve our statistical power in addressing the possibility of preferential MDV integration sites in late-stage, MD gonad lymphomas. The data indicated no evidence for a host chromosome with preferential MDV integration among gonad lymphomas. However, a low sample number (*n* = 8) in this analysis may have impacted our ability to detect slightly preferential, but not ubiquitous, sites of MDV integration in lymphoma cell populations. 

Furthermore, we sought to assess the diversity of cell populations, as defined by comprehensive and chromosome-specific MDV integration profiles, within lymphomas collected between 50–52 dpi and 60–61dpi, analyzed in study parts II-III. MD gonad lymphomas showed a decreasing trend in cellular diversity score from samples collected at 60–61 dpi as compared to 50–52 dpi. These results support a model in which tumor masses examined closer to initial infection are composed of a ‘diverse’ collection of newly-transformed lymphocytes, many of which fail to persist to create an expanded cell lineage or metastases. Thus, MD lymphomas examined after a prolonged MDV infection (≥60 dpi) develop from one or a small number of host lymphocyte transformation events that manage to persist through the selective process of tumorigenesis to become the predominant cell lineage within a tumor and produce metastasized tumors in other tissues. 

Finally, when we pooled MDV telomeric integration profile data from studies part I-III to analyze a total of 21 MD gonad lymphomas, the result indicated that there was not significantly elevated MDV integration into an individual macro- or sex chromosomes (GGA 1-4, Z or W) or into macrochromosomes, as a group, versus intermediate-sized chromosomes and microchromosomes, as groups. However, macrochromosomes had a slightly higher median MDV integration per chromosome amongst gonad lymphoma samples, suggesting that future studies with more lymphoma tissues could re-evaluate the question of biased MDV integration events in transformed host T cells.

## 5. Conclusions

In summary, we generated single-cell, cytogenomic datasets for 37 MD neoplastic tissues from unvaccinated host birds to deeply analyze the dynamics of oncogenic MDV at the level of the host genomic DNA and better understand the cellular transformation events that drive MD lymphoma development. Our viral cytogenomic profiling data supports our prior hypothesis that MDV^+^ host cells with the MDV telomere-integrated phenotype (without the presence of episomal, chromosome-associated MDV) represent transformed cells. Related to this finding, the absence of the MDV chromosome-associated only phenotype in our data indicates that neoplastic tissues of oncogenic MDV-infected birds lack dividing host cells with lytically-replicating MDV. Our temporal MDV integration profiling further suggests a model in which MD lymphomas show temporally decreasing cellular heterogeneity, as tracked by MDV integration profiles, due to selective processes in the course of infection and tumorigenesis. Finally, there is not clear evidence of a hotspot of MDV telomeric integration into a specific host chromosome(s). Thus, specific integration sites do not appear to endow a selective advantage to transformed host cells and thereby improve probability of survival and proliferation. In summary, MDV telomeric integration combined with viral oncogene expression, including *Meq*, are critical to host cell transformation, while the chromosome site of MDV integration is not. These data should serve as a resource for future studies into the role of MDV integration, infection, and latency in oncogenic transformation of host cells.

## Data Availability

All data relevant to the reported findings are available in the main text and the Supplementary Materials. Please contact the corresponding author for additional image or data requests.

## Supplementary Materials

The following are available online at https://www.mdpi.com/article/10.3390/genes12101630/s1, Table S1: Neoplastic samples used for UCD cytogenomic and USDA-ADOL driver mutation studies, Table S2: Chicken chromosome-specific BAC DNA probes applied in MDV FISH experiments.

## Appendix A

The mitotically-dividing cells of F_1_ progeny have the canonical chicken telomeric profile [61,69], in which most chromosomes possess normal terminal telomeres, but approximately three chromosomes have mega-telomeres and GGA1 contains interstitial telomeric DNA (Figure A1).
